# Decreased Arsenic Disposition and Alteration of its Metabolic Profile in mice Coexposed to Fluoride

**DOI:** 10.1007/s12011-023-03764-3

**Published:** 2023-07-14

**Authors:** Luz C. Sanchez Peña, Angel Barrera Hernández, Luz M. Del Razo

**Affiliations:** Departmento de Toxicologia, Centro de Investigación y de Estudios Avanzados, Av. IPN 2508, San Pedro Zacatenco, Mexico City, 07360 Mexico

**Keywords:** Arsenic, Fluoride, Metabolism, Disposition, Mouse, Interaction

## Abstract

Inorganic arsenic (iAs) and fluoride (iF) are ubiquitous elements whose coexistence is frequent in several regions of the world due to the natural contamination of water sources destined for human consumption. It has been reported that coexposure to these two elements in water can cause toxic effects on health, which are controversial since antagonistic and synergistic effects have been reported. However, there is little information on the possible toxicological interaction between concurrent exposure to iAs and iF on the iAs metabolism profile.

The goal of this study was to determine the effect of iF exposure on iAs methylation patterns in the urine and the tissues of female mice of the C57BL/6 strain, which were divided into four groups and exposed daily for 10 days through drinking water as follows: purified water (control); arsenite 1 mg/L, fluoride 50 mg/L and arsenite & fluoride 1:50 mg/L.

To characterize the iAs methylation pattern in concomitant iF exposure, iAs and its methylated metabolites (MAs and DMAs) were quantified in the tissues and the urine of mice was exposed to iAs alone or in combination. Our results showed a statistically significant decrease in the arsenic species concentrations and altered relative proportions of arsenic species in tissues and urine in the As-iF coexposure group compared to the iAs-exposed group. These findings show that iF exposure decreases arsenic disposition and alters methylation capacity.

Nevertheless, additional studies are required to elucidate the mechanisms involved in the iAs-iF interaction through iF exposure affecting iAs disposition and metabolism.

## Introduction

Inorganic arsenic (iAs) and fluoride (iF) are two geogenic and anthropogenic contaminants widely distributed in the environment and commonly identified in contaminated groundwater. The maximum permissible limits of iAs and iF in drinking water are 10 µg/L and 1.5 mg/L, respectively [1]. The coexistence of these elements has been reported in different parts of the world [2, 3]. including in developing countries where clean and safe surface water is scarce [4–6]. It is estimated that 300 million people are exposed to groundwater contamination by iAs and iF through drinking water [7].

The individual toxic effects of iAs and iF exposure have been widely studied and are relatively well known. The biological effects and possible interactions when the exposure is simultaneous are not a clear-cut understanding of their combined toxicity, and the results are controversial since independent, antagonistic, and synergistic effects have been reported [3].

**Fig. 1:**
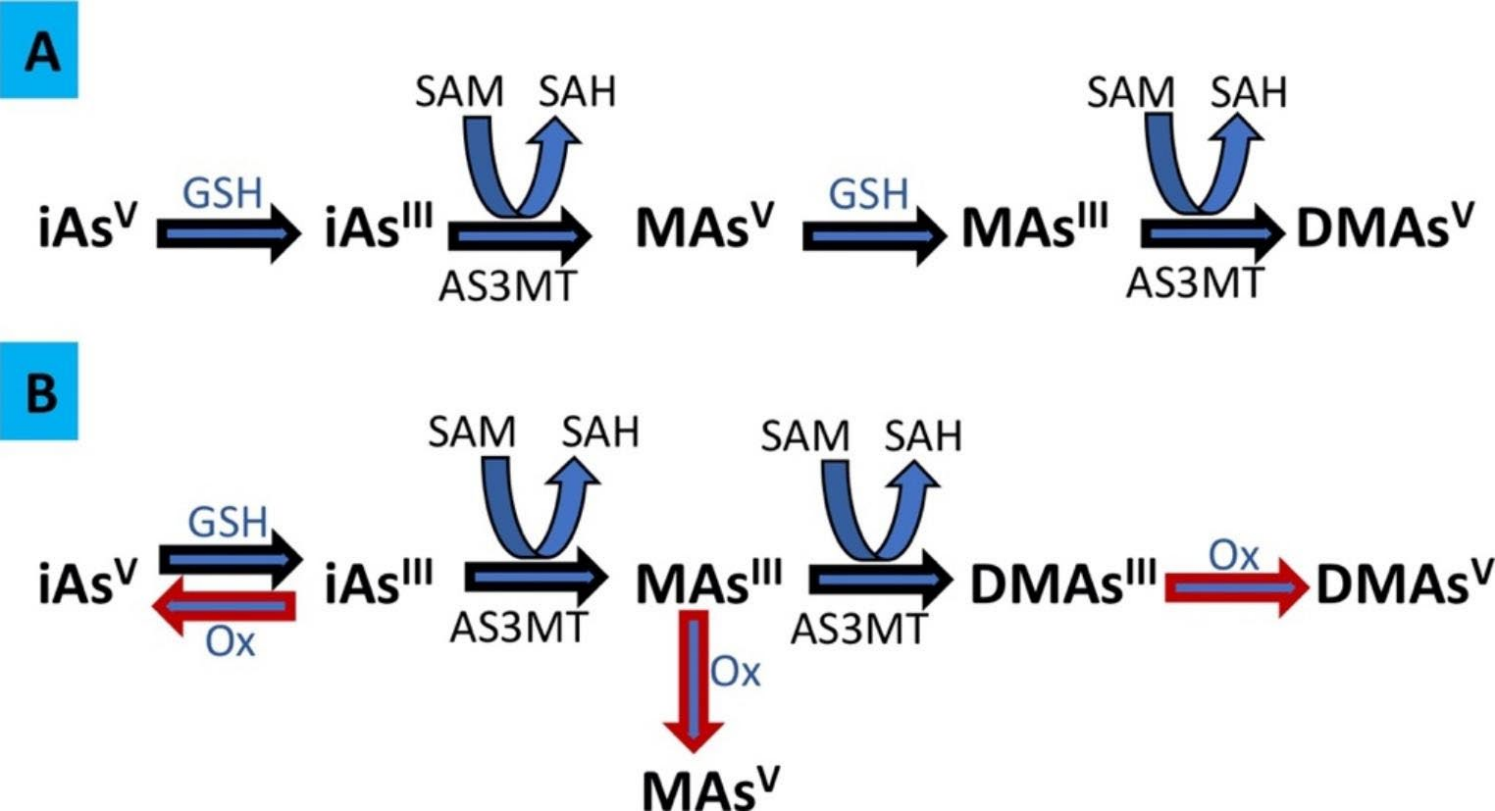
**Biotransformation of inorganic arsenic in mammals.** **A**, Conventional pathway; **B**, Recent model that describe the sequence of reactions catalyzed by AS3MT without a change in arsenical oxidation state (III). Abreviations: iAs. inorganic arsenic; GSH: reduced glutathione; AS3MT: arsenic methyltransferase; SAM:S-adenosylmethionine; SAH: S-adenosylhomocysteine; MAs: monomethylated arsenic; DMAs: dimethylated arsenic, and Ox: oxidative environment

iAs toxicity is closely related to its metabolism [8]. The conventional iAs methylation process includes reduction reactions where iAs(V) must first be reduced to iAs(III). This reduction process can be mediated by reduced glutathione (GSH), followed by a methylation process catalyzed by arsenic methyltransferase (AS3MT) with S-adenosylmethionine (SAM) as a methyl donor group where iAs is methylated to monomethylated arsenic (MAs) and dimethylated arsenic (DMAs) [9, 10]., Fig. 1A. Recent evidence suggests an arsenic methylation pathway unchanged in its oxidative state (III) and interconversion to the oxidative state of arsenic (V) in the cellular environment [11], Fig. 1B.

Evidence from a meta-analysis of epidemiological studies shows that the metabolism of iAs is influenced by several factors, such as iAs exposure magnitude, smoking, alcohol drinking, age, sex [12], and coexposure to other elements, such as selenium [13]. In a previous study, we showed the interaction between iAs and iF exposure on iAs metabolism profile alteration in a Mexican adult population exposed to low and moderate concentrations of iAs and relatively high concentrations of iF [14].

Despite the common iAs-iF coexposure, little is known about the effects of iF exposure on iAs metabolism. Therefore, the aim of this study was to evaluate the effect of iAs-iF coexposure on the methylation pattern of iAs in urine and tissues of mice coexposed to iAs-iF after exposure to AsF in drinking water.

## Methodology

### Animal Treatment

The methylation pattern of iAs and its methylated metabolites was examined in young 8-week-old C57BL/6 female mice from 15 to 17 g body weight obtained from Institutional (Centro de Investigación y de Estudios Avanzados; Cinvestav-IPN). Mice were housed in plastic cages with shavings as bedding material. The room was kept on a 12/12-h light/dark cycle at a temperature of 22 ± 1 °C, humidity of 40–60%, filtered air with a 95% efficiency and noise level lower than 85 dB. Mice were provided with free access to rodent food containing 0.147 mg As/kg and 12 mg F/kg (PicoLab® Mouse Diet 20, #5058, LabDiet®; Haward, CA) and purified water containing 1 µg/L iAs and 10 µg/L iF.

The procedures were approved by the Institutional Animal Care and Use Committee (CICUAL, Protocol#0277 − 18) in accordance with the Mexican Guideline Regulations of Animal Care and Maintenance [15] and the international guidelines for the use and care of laboratory animals as adopted and promulgated by the US National Institutes of Health. After 8 days of acclimatization, thirty-two mice were randomly assigned to four experimental groups (n = 8) as follows: control (received purified water), iF (fluoride exposure received water containing sodium fluoride at 50 mgF/L), iAs (arsenic exposure received water containing sodium arsenite at 1 mgAs/L), and group iAs-iF (arsenic & fluoride 1: 50 mg/L) through drinking water for 10 days. The sodium arsenite and sodium fluoride (Sigma‒Aldrich, 99% pure) used to prepare the solutions to expose the iAs, iF and iAs-iF groups were prepared daily. Body weight, water intake and food consumption were monitored every day. For urine collection, twelve hours prior to euthanasia, two mice from each group were housed in metabolic cages (Nalgene Co., Rochester, NY). Mice were maintained with free access to pellet rodent chow and tap water while in metabolic cages. Urine samples were collected for 12 h. At the time of euthanasia, each mouse was anesthetized using ketamine and xylazine. Kidney, liver, lung and urine bladder were extracted and washed in ice-cold isotonic saline solution to remove debris and blood, and urine and tissues were frozen at 80 °C until analysis.

### Arsenic Analysis

20% tissue homogenates (w/v) were prepared in deionized water on ice. Tissue homogenates and urine were used to measure the concentration of iAs and its methylated metabolites MAs and DMAs by hydride generation-cryotrapping-atomic absorption spectrometry using a Perkin Elmer Analyst 400 spectrometer (Perkin Elmer, Norwalk, CT), as described by [16]. The limits of detection (LOD) for iAs, MAs, and DMAs using this method are 0.12, 0.13, and 0.14 ng As, respectively. Urine standard reference material (SRM) 2669 level 1 and level 2 from the National Institute of Standard and Technology (NIST) were used as quality controls to validate the analysis of the arsenic species at low and high concentrations, respectively. Accuracy ranged from 91 to 101% with a variation coefficient between 0.5 and 10% in duplicate samples.

### Indicators of iAs Metabolism

Arsenic species and total arsenic, TAs (sum of iAs, MAs, and DMAs), were reported as ng/g. The relative proportions of arsenic species of iAs%, MAs%, and DMAs% were calculated by dividing the amounts of each arsenic species by that of TAs, considering the sum of arsenicals as 100% in the denominator.

Arsenic methylation indices were calculated as MAs/iAs (primary methylation index) and DMAs/MAs (secondary methylation index).

### Statistical Analysis

All statistical analyses were performed using procedures available in GraphPad Prism version 8.0 (Boston’s MA). One-way analysis of variance with Student-Newman-Keuls or Tukey’s multiple comparison post-test was used to assess differences in the concentrations and proportions of As species. Differences with p less than 0.05 (p < 0.05) were considered statistically significant.

## Results

### Estimated Daily Amount of iAs Ingested

Average daily iAs intake in drinking water per mouse was estimated based on the water consumption. Mice in the iAs group ingested 4.33 µg of iAs/day while iAs-IF group ingested 4.26 µg/day (Table 1). the estimated daily amount of iAs ingested in iAs and iAs-IF groups were similar.

**Table 1:** Estimated intake of inorganic arsenic by mouse

Parameters	Control	Fluoride (iF)	Arsenic (iAs)	Arsenic-Fluoride (iAs-iF)	iAs vs. iAs-iF p-value
**Water consumption mL/day**	4.14 (4.08–4.61)	4.11 (4.02–4.37)	4.33 (4.19–4.42)	4.26 (4.11–4.35)	0.124
**Food consumption g/3 days**	6.33 (5.79–6.73)	6.08 (5.33–8.75)	6.09 (5.30–8.80)	6.29 (6.02–6.99)	0.668
**Intake of arsenic µg/day**	0.004 (0.004–0.005)	0.004 (0.004–0.004)	4.33 (4.19–4.43)	4.26 (4.11–4.35)	0.124

Mice received purified water (control), iF (50 mg/L), iAs(1 mg/L), and iAs & iF (1:50 mg/L) via drinking water for 10 days Results were expressed as median (25% percentile – 75% percentile), (n = 8). Comparative water and food consumption, and calculated intake of inorganic arsenic between iAs and iAs-iF groups by Mann Whitney test

### Internal Arsenic Dosimetry and Urinary Excretion Level

Arsenic in the form of iAs, MAs and DMAs was quantified in the kidney, liver, lung and urine bladder of mice in the experimental groups. As expected, DMAs were the predominant species in all tissues of the study groups. As expected, the concentrations of arsenicals in the control and iF groups were significantly lower than those presented in the groups exposed to iAs and the combination of iAs-iF. Since our basic interest is the comparison between iAs vs. iAs-F groups, the statistical analysis was focused on this comparison. The concentrations of iAs, MAs and DMAs in the kidney, liver, lung, and urinary bladder were lower in the coexposed iAs-iF group than in the iAs group. This decrease in the internal dose of these organs was significant for the liver and kidney and marginally significant for the lung, whereas in the urinary bladder, it was only marginally significant for MA levels. (Table 2). The sum of arsenicals/iAs + MAs + DMAs) represents the internal level of toxic arsenic in each tissue after 10 days of daily exposure. It was in the order of urine bladder > lung > liver > kidney (Table 2).

**Table 2:** Concentrations of arsenic species and sum of arsenicals in mouse tissues

Arsenic in Tissue	Control	Fluoride (iF)	Arsenic (iAs)	Arsenic-Fluoride (iAs-iF)	iAs vs. iAs-iF p-value
**Kidney, ng/g**					
iAs	0.25 ± 0.01	0.14 ± 0.01	4.76 ± 0.68	3.25 ± 0.24	**0.048**
MAs	≤ LD	≤ LD	1.47 ± 0.24	0.75 ± 0.16	**0.021**
DMAs	0.63 ± 0.03	1.49 ± 0.27	40.32 ± 5.61	20.24 ± 1.28	**0.002**
Sum As species	1.03 ± 0.03	1.77 ± 0.27	46.55 ± 6.40	23.64 ± 1.52	**0.003**
**Liver, ng/g**					
iAs	1.10 ± 0.09	0.16 ± 0.03	9.26 ± 1.96	3.59 ± 0.49	**0.005**
MAs	0.22 ± 0.02	≤ LD	7.30 ± 1.26	3.01 ± 0.51	**0.003**
DMAs	0.48 ± 0.04	2.03 ± 0.22	39.98 ± 3.47	21.63 ± 1.52	**< 0.0001**
Sum As species	1.80 ± 0.12	2.34 ± 0.23	56.54 ± 6.29	28.24 ± 2.02	**0.0003**
**Lung, ng/g**					
iAs	0.16 ± 0.02	0.16 ± 0.17	2.05 ± 0.26	1.32 ± 0.28	0.067
MAs	0.17 ± 0.01	0.16 ± 0.02	1.25 ± 0.15	0.81 ± 0.17	0.070
DMAs	0.59 ± 0.03	3.37 ± 0.77	68.69 ± 5.80	51.88 ± 6.23	0.062
Sum As species	0.91 ± 0.05	4.11 ± 0.85	71.88 ± 5.99	54.01 ± 6.49	0.055
**Urinary Bladder, ng/g**					
iAs	≤ LD	≤ LD	10.95 ± 3.64	7.51 ± 2.90	0.386
MAs	9.62 ± 4.13	10.21 ± 4.58	1.62 ± 0.75	0.14 ± 0.01	**0.051**
DMAs	11.82 ± 5.25	12.24 ± 5.28	602.0 ± 114.1	344.0 ± 76.65	0.084
Sum As species	20.01 ± 8.13	25.44 ± 11.96	644.5 ± 138.9	368.0 ± 96.42	0.386

Abbreviatures: iAs: inorganic arsenic, MAs: monomethyl arsenic specie; DMAs: dimethyl arsenic specie leves. LD: Limit of detection = 0.13 ng MAs. Mice received purified water (control), iAs(1 mg/L), iF (50 mg/L) and iAs & iF (1:50 mg/L) via drinking water for 10 days. Results are expressed as mean ± standard error (n = 8). Comparative concentrations between iAs and iAs-iF groups by Student’s t-test.

Because ingested arsenic is mainly excreted by urine, the concentrations of iAs and its metabolites were determined at the end of exposure. To collect enough urine sample (~ 0.4 mL), two mice were placed per metabolic cage; the results were expressed in ng/mL. Urinary DMAs and the sum of As species were lower in the concurrent exposure group than in the iAs group exposed alone (Table 3).

The two groups that were not exposed to iAs (control and exposed to iF) presented low concentrations of arsenicals in both tissues and urine, possibly due to trace iAs concentrations of the food.

**Table 3:** Concentrations of arsenic species and sum of arsenicals in urine

Arsenic Specie ng/mL	Control	Fluoride (iF)	Arsenic (iAs)	Arsenic-Fluoride (iAs-iF)	iAs vs. iAs-iF p-value
iAs	1.80 ± 0.67	1.67 ± 0.56	20.61 ± 1.90	31.96 ± 5.99	**0.015**
MAs	≤ LD	≤ LD	2.56 ± 0.43	3.99 ± 0.40	**0.009**
DMAs	35.0 ± 6.54	29.62 ± 6.26	478,25 ± 76,11	369.80 ± 35.46	**0.0416**
Sum As species	37.11 ± 6.32	31.40 ± 7.18	501.42 ± 77.37	405.75 ± 35.59	0.065

Abbreviatures: iAs: inorganic arsenic, MAs: monomethyl arsenic specie; DMAs: dimethyl arsenic specie levels. LD: Limit of detection = 0.13 ng for MAs. Mice received purified water (control), iAs (1 mg/L), iF (50 mg/L) and iAs & iF (1:50 mg/L) via drinking water for 10 days. Results are expressed as mean ± standard deviation (n = 4). Comparative concentrations between iAs and iAs-iF groups by Student’s t-test.

### Arsenic Methylation Profile

**Fig. 2:**
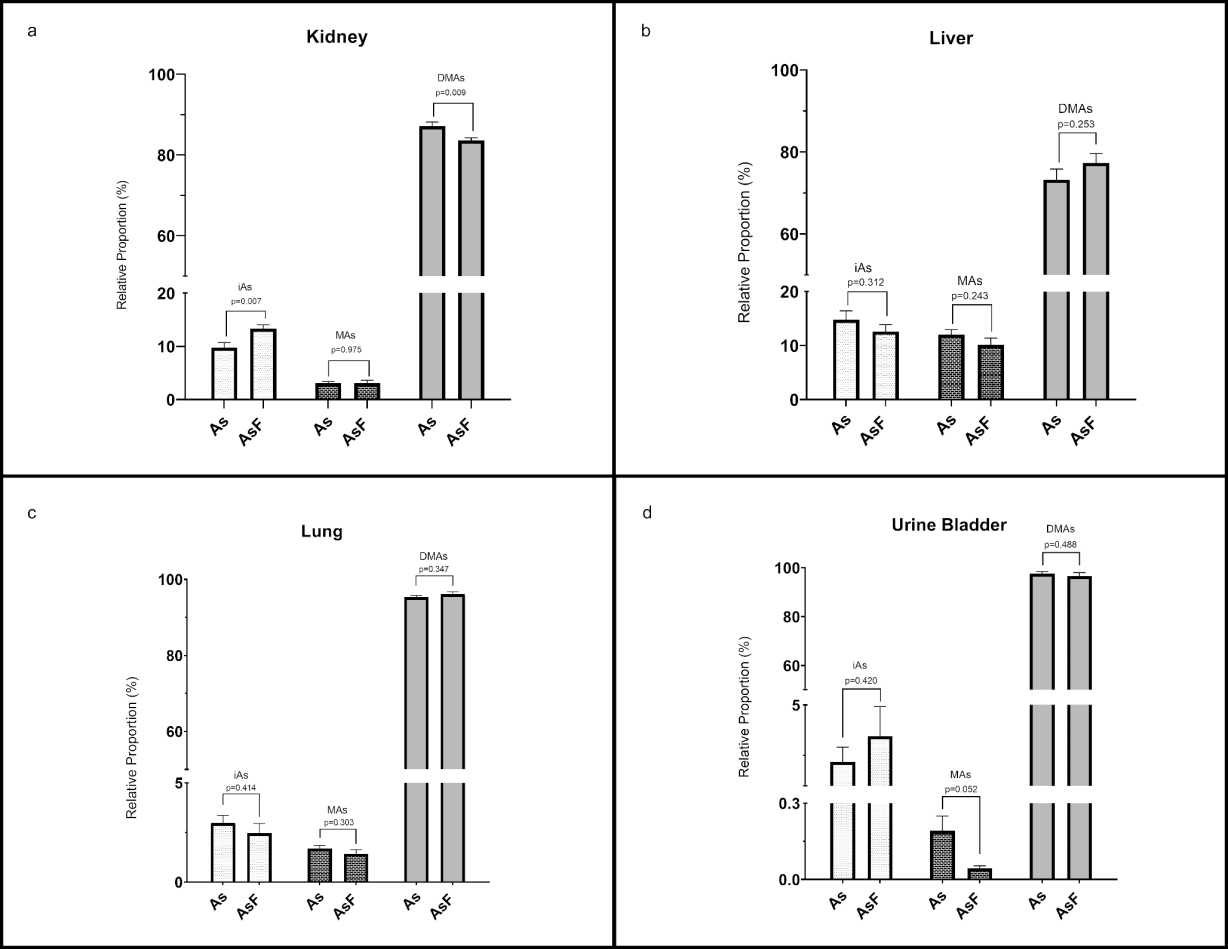
**Relative proportion of arsenicals in tissues.** Arsenic specues (iAs inorganic arsenic, MAs monomethyl-arsenic metabolite and DMAs. dimethyl-arsenic metabolite) in kidney (**a**), liver (**b**), lung (**c**) and urine bladder (**d**) of mouse exposed to iAs. arsenite (1 mg/L) and As-iF [arsenite (1 mg/L) & fluoride (50 mg/L)] via drinking water for 10 days. The relative proportions of arsenical species were expressed as a percentage (mean ± standard error; n = 8). Differences between iAs exposed group vs. combined iAs-iF exposed group were evaluated by Student t test.

**Fig. 3:**
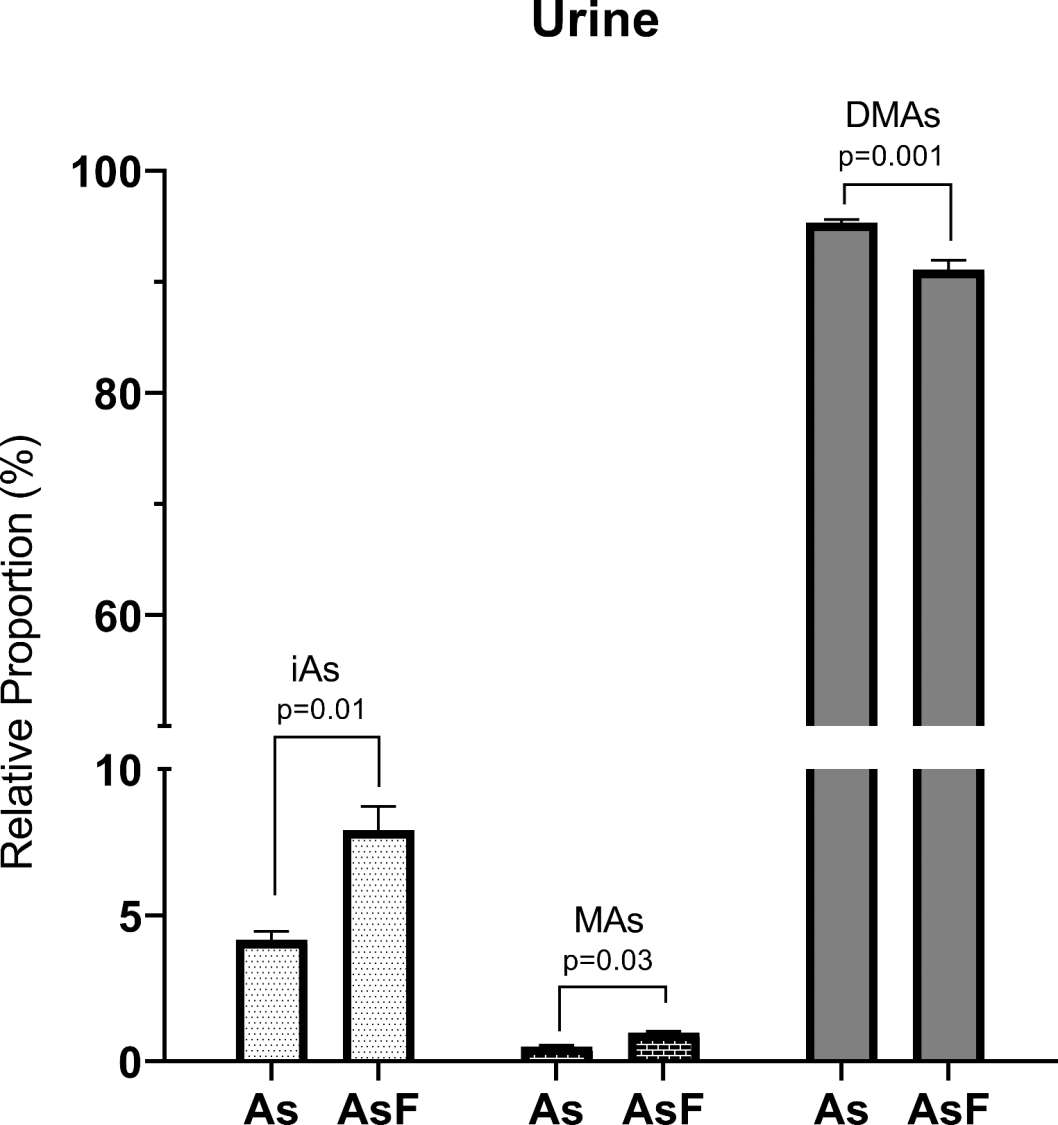
**Relative proportion of arsenicals in urine.** Arsenic species (iAs inorganic arsenic, MAs monomethyl-arsenic metabolite and DMAs. dimethyl-arsenic metabolite) in urine of mouse exposed to iAs. arsenite (1 mg/L) and iAs-iF [arsenite (1 mg/L) & fluoride (50 mg/L)] via drinking water for 10 days. The relative proportions of arsenical species were expressed as a percentage (mean ± standard error; n = 4). Differences between iAs exposed group vs. combined iAs-iF exposed group were evaluated by Student t- test.

In relative terms, DMAs were the most abundant metabolites in the lung, urinary bladder, and urine (~ 92%) and in the liver and kidney (~ 79%) (Fig. 2). There were marked differences in arsenic speciation profiles in kidney ). Altered profiles of %iAs and %DMAs were observed in the kidney depending on iF coexposure (Fig. 2a). Arsenic speciation in urine shows a lower relative proportion of DMAs with an increase in iAs and MAs (Fig. 3).

Table 4 shows the first (MAs/iAs) and second (DMAs/MAs) methylation indices obtained in tissues. Only the secondary methylation index in the liver was significantly higher in the coexposure group iAs-iF than in the iAs exposure group. For the urinary pattern of arsenicals, DMA/MAs were also altered in the case of coexposure to iF (Table 5),

**Table 4:** Arsenic methylation indices in mouse tissues

Arsenic Methylation Indices	Control	Fluoride iF	Arsenic iAs	Arsenic-Fluoride iAs-iF	iAs vs. iAs-iF p-value
**Kidney**					
MAs/iAs	0.57 ± 0.02	1.07 ± 0.06	0.38 ± 0.08	0.25 ± 0.05	0.192
DMAs/MAs	4.3 ± 0.20	10.64 ± 1.94	39.54 ± 11.92	45.97 ± 12.93	0.718
**Liver**					
MAs/iAs	0.21 ± 0.024	1.05 ± 0.08	0.85 ± 0.04	0.82 ± 0.06	0.709
DMAs/MAs	2.23 ± 0.046	14.51 ± 1.09	6.74 ± 0.52	8.84 ± 0.93	**0.039**
**Lung**					
MAs/iAs	1.10 ± 0.05	0.69 ± 0.15	0.64 ± 0.07	0.96 ± 0.25	0.220
DMAs/MAs	3.59 ± 0.22	22.90 ± 5.62	61.75 ± 6.28	88.70 ± 14.76	0.107
**Urine Bladder**					
MAs/iAs	102.1 ± 38.62	94.51 ± 35.58	0.20 ± 0.12	0.35 ± 0.21	0.537
DMAs/MAs	1.83 ± 1.20	2.05 ± 1.14	1835 ± 901	2495 ± 684.3	0.579

Abbreviatures: iAs: inorganic arsenic, MAs: monomethyl arsenic specie; DMAs: dimethyl arsenic specie. Mice received purified water (control), iAs (1 mg/L), iF: fluoride (50 mg/L) and iAs & iF (1: 50 mg/L) via drinking water for 10 days. Results were expressed as mean ± standard error (n = 8). Comparative arsenic methylation indices between arsenic and arsenic  & fluoride groups by Student t test.

**Table 5:** Arsenic methylation indices in urine

Arsenic Methylation Indices	Control	Fluoride iF	Arsenic iAs	Arsenic-Fluoride iAs-iF	iAs vs. iAs-iF p-value
MAs/iAs	0.07 ± 0.012	0.027 ± 0.061	0.125 ± 0.013	0.127 ± 0.007	0.9312
DMAs/MAs	365.77 ± 36.42	296.2 ± 34.78	188.20 ± 12.85	93.18 ± 5.87	**0.027**

Abbreviatures: iAs: inorganic arsenic, MAs: monomethyl arsenic specie; DMAs: dimethyl arsenic specie. Mice received purified water (control), iAs (1 mg/L), iF: fluoride (50 mg/L) and iAs & iF (1: 50 mg/L) via drinking water for 10 days. Results were expressed as mean ± standard error (n = 8). Comparative arsenic methylation indices between arsenic and arsenic + fluoride groups by Student t test.

## Discussion

This study evaluated the distribution and excretion of arsenicals in a mouse model at biologically relevant concentrations, where arsenic disposition and excretion are shown to be lower in the concurrent exposure with iF than when exposed only with iAs.

The C57BL/6 mouse strain was chosen as an experimental model, as it is characterized by being a good model for exposure and metabolic effects of exposure to iAs in addition to being sensitive to the effects of exposure to iF [9, 17, 18].

Differences in iF toxicokinetics have been reported between rodents and humans. In rodents, iF is distributed and cleared 10 times faster, and the effects are less toxic [19, 20]. Thus, in our work, mice were exposed to concentrations of 50 ppm by drinking water and considering that this concentration would equal concentrations of 5 ppm, rodents were exposed to a concentration that can be found naturally in water contaminated with iF [5]. Regarding the concentrations of iAs we used 1 mg/L, it was considered that in rodents, in the same way as iF, iAs clearance is more efficient than in humans, making them less susceptible [21]. These concentrations were used to simulate iAs concentrations found in endemic populations of 0.2 mg/L. There are many regions with concentrations of iAs in water of 0.2 mg/L [5]. The iF:iAs ratio used in the coexposed group was 50 times (50:1).

Several experimental studies in rodents have recently been conducted with the combined exposure of iAs and iF, evaluating the effect of iAs and iF, individually or together, on oxidative stress, inflammation, and reproductive and neurotoxicological effects, and they concluded that the simultaneous administration of iAs and iF is less toxic [22–25]. A limitation in these studies is that they used high concentrations of the same exposure magnitude for both pollutants when it is known that iF is usually in concentrations 50 to 150 times higher compared to iAs.

We observed that - iF could cause an antagonistic effect due to the lower concentration of iAs and its methylated metabolites in liver, kidney, and urine of mice coexposure to iAs-iF than those observed with exposure to iAs. Interestingly, other authors have reported a lower disposition of As in the presence of iF. The mean total As levels in rat liver were 1.2 and 0.8 µg/g in groups exposed to iAs (50 mg/L) and iAs-iF (50–50 mg/L) for 9 months, respectively [26]. The total arsenic level was 0.2 µg/g vs. 0.1 µg/g in zebrafish brains exposed to iAs alone (0.050 mg/L) and iAs-iF (0.050-15 mg/L) for 60 days, respectively [27].

The possibility exists that iAs uptake may be decreased in the presence of iF and thus explaining the decrease in arsenical concentrations in urine and mouse tissue in coexposure to iF. Both pollutants are known to be absorbed in the intestine through both paracellular (tight junctions) and transcellular mechanisms [26, 28–30]. Therefore, a possible competitive interaction is suggested in the absorption in the intestine since both contaminants share both routes.

Some authors have suggested the formation of iAs-iF complexes, since iF, being an electronegative element, can form ionic bonds with iAs and form arsenic trifluoride (AsF_3_), a complex that is not easily absorbed by the intestine [31], while in its pentavalent state, it can form arsenic pentafluoride (AsF_5_) by decreasing the ionization of iAs [32]. Although salts of AsF_3_ and AsF_5_ have been synthesized, there is no evidence of these salts formation in water or in vivo during the administration of iAs and iF and consequently of their possible presence or in gastric transit.

Additionally, it has been shown that both iAs and iF can modify populations of the gastrointestinal microbiota [33, 34]. Among the modifications that both contaminants share by the microbiota is the reduction of the gastrointestinal barrier and the damage to the tight junctions of the intestine, which leads to permeability alteration [35].

We propose that iF with this competitive interaction can modify the absorption of iAs, thus distributing its concentration toward different organs, such as the kidney and liver, which were evaluated in the present study.

On the other hand, the evaluation of the metabolic profile is important. Differences in the concentrations or proportions of iAs, MAs, and DMAs have been linked to the susceptibility of a variety of adverse health effects of iAs exposure [36, 37]. To our knowledge, this is the first in vivo experimental study that shows the interaction effect of iAs-iF coexposure on the methylation pattern of iAs and its methylated metabolites in tisúes, and urine mice coexposed to iAs-iF through drinking water. The methylation arsenic profile in the liver, urine bladder, and lung was not altered by coexposure but altered by distinct organ-specific differences in the distribution and methylation of iAs and its metabolites, which have been reported [38].

Alteration of the iAs methylation pattern may be influenced by GSH depletion reported by iF exposure [39]. It has already been shown that GSH depletion could be a potential mechanism associated with altered methylation capacity [17, 40].

Interestingly, the lower expression (1.8 times) of the *as3mt* gene was recently identified in the brain of zebrafish coexposed to iF (15 ppm) and iAs (0.05 ppm) compared to exposure to iAs alone, which helps explain the alteration in iAs metabolic pattern [27].

## Conclusions

A decreased disposition of arsenicals and altered methylation profile were observed in concurrent iAs-iF exposure, suggesting that iF exposure plays an important role in iAs metabolism. Further studies are required to determine the mechanism by which iF exposure affects iA metabolism and better characterizes the health risks associated with combined exposure to iAs and iF.

## Data Availability

The datasets generated during and/or analyzed during the current study are available from the corresponding author on reasonable request.

## Abbreviations

iAs: Inorganic arsenic
iF: Inorganic fluoride
MAs: Monomethylated arsenic metabolite
DMAs: Demethylated arsenic metabolite
GSH: Glutathione
AS3MT: Arsenic methyltransferase
SAM: S-adenosylmethionine
